# EM biofertilizer and organic fertilizer co-application modulate vegetation-soil-bacteria interaction networks in artificial grasslands of alpine mining regions

**DOI:** 10.3389/fmicb.2025.1659475

**Published:** 2025-09-18

**Authors:** Liangyu Lyu, Jianjun Shi, Pei Gao, Zongcheng Cai, Fayi Li

**Affiliations:** ^1^Academy of Animal Husbandry and Veterinary Sciences, Qinghai University, Xining, China; ^2^Key Laboratory of Adaptive Management of Alpine Grassland, Xining, China; ^3^State Key Laboratory of Ecology and Plateau Agriculture and Animal Husbandry in Sanjiangyuan Jointly Established by the Ministry of Provincial Affairs, Qinghai University, Xining, China

**Keywords:** Muli mining area, EM biofertilizer, sown pasture, soil physicochemical properties, bacterial community structure

## Abstract

To address vegetation establishment challenges caused by poor soil in the alpine mining areas of the Qinghai–Tibet Plateau. Through three-year field experiments, this study systematically investigated the effects of combined EM microbial fertilizer and organic fertilizer application on vegetation community characteristics, soil physicochemical properties, and bacterial community diversity/functional structure in artificial grasslands of alpine mining areas. Key findings include: (1) The synergistic treatment of EM biofertilizer and organic fertilizer significantly improved the physical and chemical properties of soil and the characteristics of vegetation community. The Y2E2 treatment consistently enhanced vegetation community characteristics and soil physicochemical metrics in 2023 and 2024. Compared to the control (CK), it increased soil total nitrogen (TN) by 68.92 and 76.31%, reduced pH by 8.31 and 11.11%, and boosted biomass by 75.97 and 84.02%, confirming its efficacy in alleviating nutrient stress and promoting plant growth. (2) Microbiome analysis revealed that biofertilizer treatments significantly improved soil bacterial community structure. The Y2E2 and Y2E3 showed the highest OTU numbers (2,481 and 2,501 respectively). The Y2E3 increased relative abundance of Actinomycetota (+18.2%) and Acidobacteria (+12.7%) compared to CK (organic fertilizer 0.00 kg m^−2^ + EM biofertilizer 0.00 kg m^−2^), while reducing Pseudomonadota (−14.3%). The Y2E2 improved Shannon (2.36%), Ace (6.44%), and Chao1 (5.05%) indices versus CK. Y1E1 exhibited 67.11% positive correlations in microbial co-occurrence networks. (3) Environmental drivers and functional activation: Mantel tests and RDA revealed soil electrical conductivity (SEC) and pH were negatively correlated with bacterial diversity indices (except Simpson). Other soil physical and chemical indexes and plant community indexes are positively correlated with soil bacterial diversity index except Simpson index. Soil pH emerged as the key driver of bacterial community construction. Combined fertilization neutralized alkalinity, activated manganese-oxidizing and photosynthetic microbes, while excessive application triggered heterotroph competition. In summary, the combined application of EM microbial fertilizer and organic fertilizer accelerates biomass accumulation in plant communities by regulating soil pH and improving the structure, function, and diversity of soil bacterial communities. These ecological processes involving plant-soil-microbe interactions expedite the restoration of ecological functions in artificial grasslands within alpine mining areas. Among the treatments, Y2E2 demonstrated the best performance, with an application rate of 600.00 kg of EM biofertilizer per hectare combined with 20.00 tons of organic fertilizer.

## 1 Introduction

The Muli mining area, located at the southern foot of the Qilian Mountains and adjacent to Qilian Mountains National Park, serves as the headwater region of the Datong River, a major tributary of the Yellow River (Duan et al., 2022; Lyu et al., 2024). Prolonged open-pit mining activities have led to severe vegetation degradation, soil structure disruption, and the formation of extensive exposed slag areas, posing significant threats to regional ecological security and the sustainable development of grassland animal husbandry (Zhao et al., 2025). Against this backdrop, ecological restoration in the mining area has become an urgent priority. The establishment of artificial grasslands is a critical approach for ecological restoration in alpine mining regions. However, limiting factors such as poor soil organic matter content and low microbial activity severely hinder vegetation rehabilitation (Liu et al., 2024; Hu et al., 2024). While traditional chemical fertilizers can temporarily enhance soil fertility, their associated issues—such as soil acidification, compaction, and reduced microbial diversity—undermine the sustainability of restoration goals (Zhang et al., 2024; Yu et al., 2025a, 2025b). Therefore, research on green fertilization technologies that regulate soil microbial communities has emerged as a vital breakthrough in addressing the ecological restoration challenges of alpine mining areas.

The collaborative remediation technology combining microbial fertilizer and organic fertilizer has garnered considerable attention due to its ecological compatibility and functional synergism (Yan et al., 2024; Yin et al., 2019; Zeng et al., 2016). Effective microorganisms (EM) microbial fertilizer contains over 80 functional microbial groups, including photosynthetic bacteria, lactic acid bacteria, and actinomyces. It regulates rhizosphere microbial processes through multiple pathways such as organic matter decomposition, nutrient activation, and microecological regulation, thereby improving soil environment and subsequently influencing plant growth (Liu et al., 2025; Sahu et al., 2023). Zeng et al. (2016) demonstrated that EM microbial fertilizer enhances catalase activity in flue-cured tobacco rhizosphere soil and promotes plant biomass accumulation. Yin et al. (2019) and Yan et al. (2024) confirmed that combined application of EM microbial fertilizer and organic fertilizer can reconstruct soil-microbe interaction networks, increasing the relative abundance of beneficial microbial communities. Zhang et al. (2024) found that EM microbial fertilizer regulates crop yield by suppressing pathogenic bacteria abundance and enhancing microbial α-diversity and network complexity. While existing studies on combined EM microbial fertilizer and organic fertilizer application have predominantly focused on plain agroecosystems (Yan et al., 2024; Yin et al., 2019; Zeng et al., 2016), nutrient addition rates vary significantly across different study regions. In alpine mining areas, there is currently no unified fertilizer application range for the restoration of artificially restored grasslands through the co-application of microbial fertilizer and organic fertilizer. Meanwhile, the impact of fertilization on grasslands may involve a response threshold—beyond which, increased fertilizer application could lead to slowed grassland productivity growth, or even phenomena such as species decline, biodiversity reduction, and productivity decrease (Lv et al., 2025). Therefore, determining the optimal nutrient addition level by co-applying microbial fertilizer at varying dosages based on organic fertilizer application is crucial. This approach not only avoids resource waste and enhances economic efficiency but also plays a vital role in maintaining the stability of alpine mining area grassland ecosystems and promoting their sustainable development (Lyu et al., 2025).

**Figure 1:**
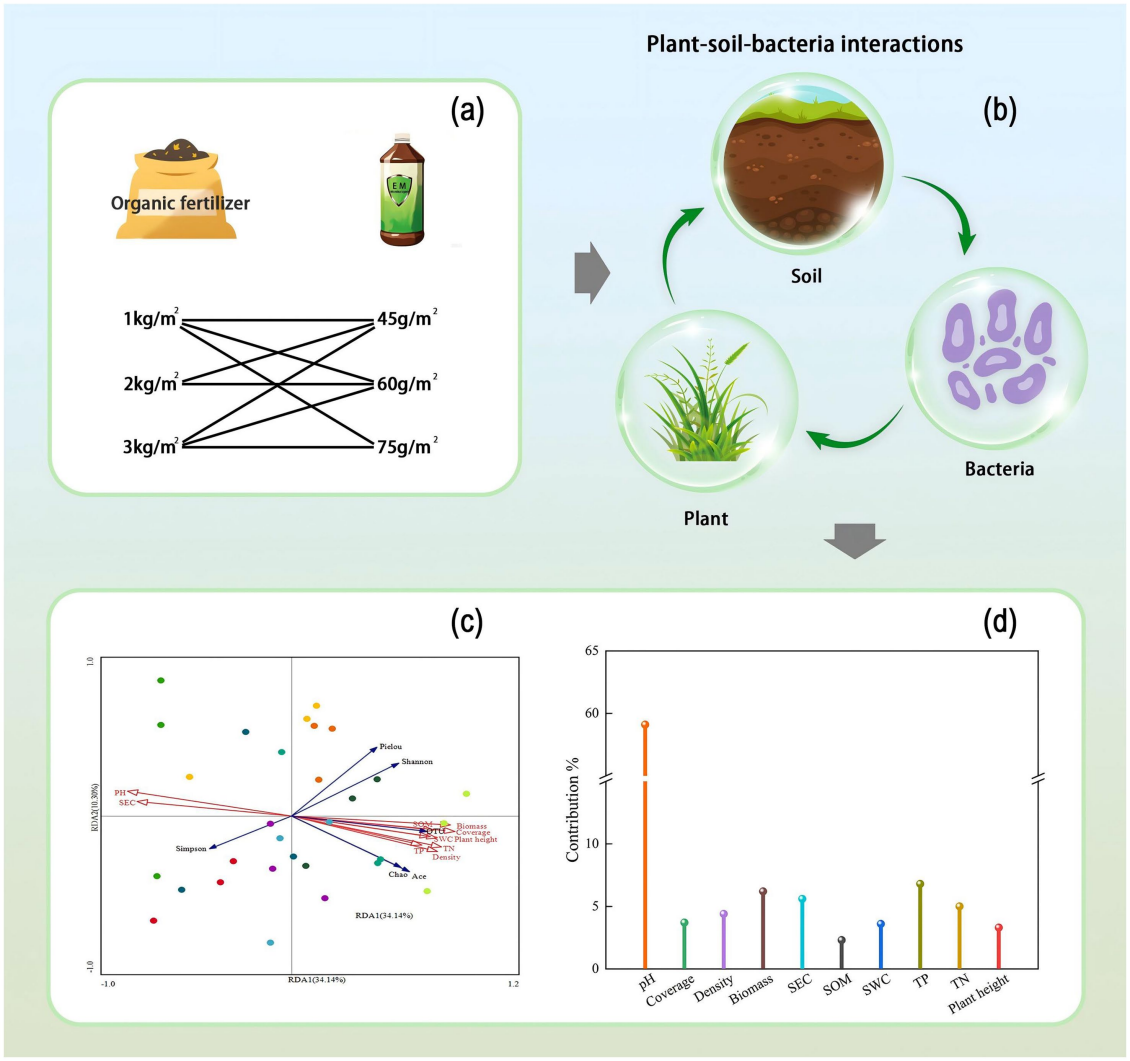
Graphic summary. **(a)** Fertilizer application rates, **(b)** Interactions among plants, soil, and bacteria, **(c)** RDA analysis, **(d)** Contribution rates of plant and soil factors.

In summary, the current study has two major limitations: (1) The research scope is confined to traditional agricultural systems (Yan et al., 2024; Yin et al., 2019; Zeng et al., 2016), lacking validation of applicability in extremely degraded habitats (e.g., alpine mining areas); (2) Functional analysis focuses on single elements (e.g., microorganisms or vegetation) (Yan et al., 2024; Yin et al., 2019; Zeng et al., 2016), neglecting coupled analysis of soil-plant-microbe systems. Moreover, mechanistic explanations remain at the phenomenological description stage. In extremely degraded ecosystems like alpine mining areas characterized by extremely infertile soil and low vegetation establishment success rates, the interaction mechanisms among microbial community structure, soil functions, and vegetation restoration remain unclear. To address the aforementioned limitations, we propose the hypothesis that combined application of EM bacterial fertilizer and organic fertilizer could enhance ecosystem multifunctionality by regulating soil bacterial community structure and function, thereby facilitating soil amelioration and vegetation restoration (Figure 1). Therefore, this study focuses on artificially established grasslands in alpine mining areas as the research object. Through a three-year field experiment, we systematically investigate the effects of combined EM bacterial fertilizer and organic fertilizer application on vegetation community characteristics, soil physicochemical properties, and soil bacterial community structure and function in alpine mining areas. The study prioritizes elucidating two scientific questions: (1) The variation patterns of soil bacterial community composition and co-occurrence network characteristics under different nutrient addition levels in alpine mining areas; (2) The interactions between bacterial community structure and soil-vegetation factors. The research findings will provide a scientific basis for ecological restoration in alpine mining areas and sustainable grassland management techniques based on soil microecology regulation.

## 2 Materials and methods

### 2.1 Study area overview

**Figure 2:**
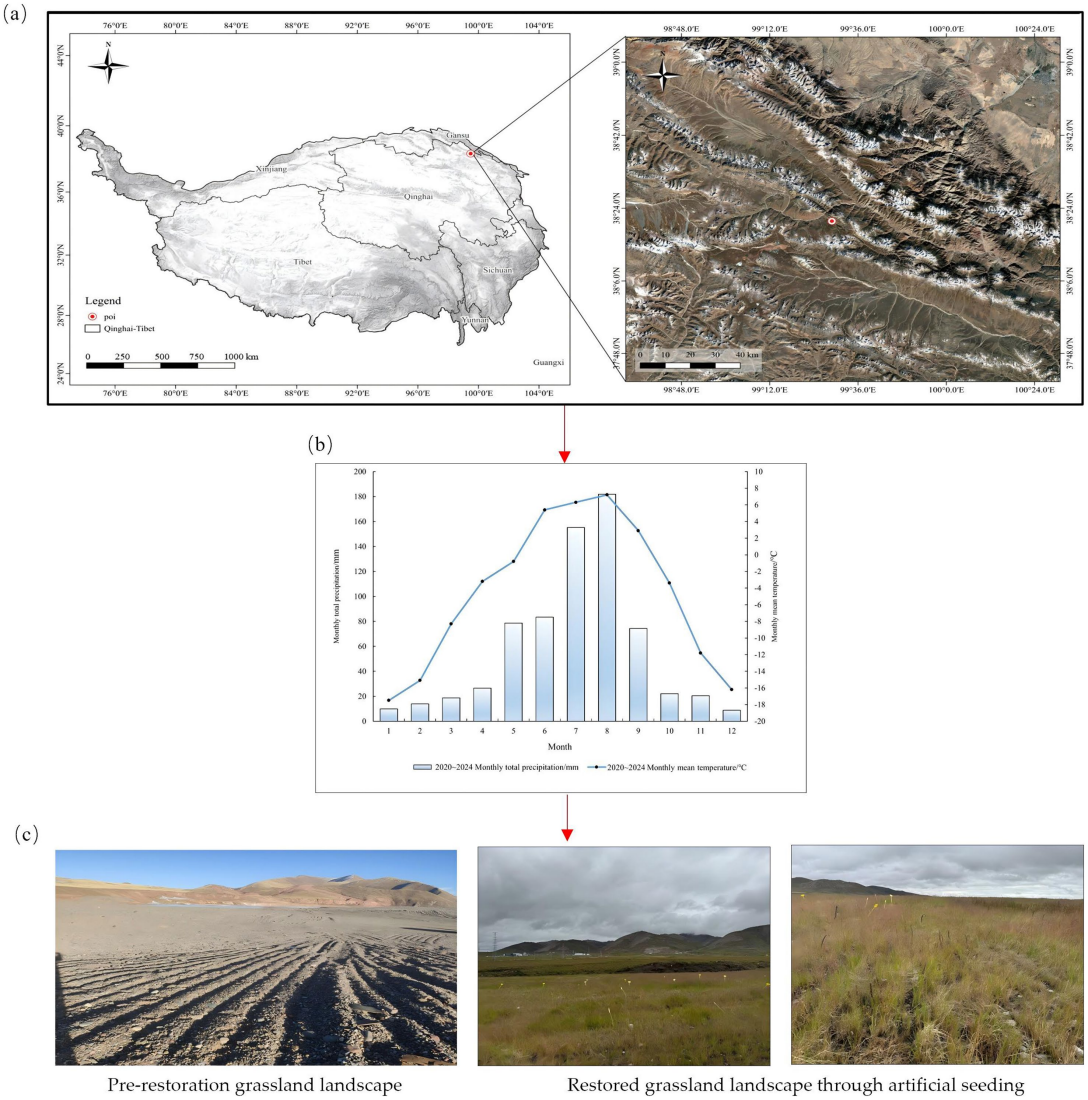
Study area, experimental site, and plot design. **(a)** Geographic location of the experimental site, **(b)** monthly average temperature and precipitation at the experimental site from 2020 to 2024 (meteorological data sourced from the Tianjun National Weather Station), **(c)** landscape before and after seeding.

The study area was located in Muli Town, Tianjun County, Qinghai Province (38°09′34″N, 99°09′40″E), with an average elevation of 4,200 meters. The region experiences long winters without summers, with the cold season lasting 7–8 months, significant diurnal temperature variations, and a semi-arid plateau climate. The annual average sunshine duration is 2,160 h, the annual average temperature was −5.3 °C, and the annual average precipitation is 626 mm, with a growing season of only 120 days (Lyu et al., 2024; Hu et al., 2024). The soil is classified as alpine meadow soil, containing significant coal gangue and rock fragments. The soil total nitrogen content is 1.05 g kg^−1^, total phosphorus content is 0.84 g kg^−1^, organic matter content is 60.34 g kg^−1^, electrical conductivity is 19,090 μS cm^−1^, pH is 8.50, and moisture content is 15.0%. The original vegetation type is alpine meadow, dominated by *Carex parvula* O. Yano, *Carex alatauensis* S. R. Zhang, and *Elymus nutans* Griseb. An overview of the study site is shown in Figures 2a,b.

### 2.2 Experimental design

The microbial biofertilizer used was a composite effective microorganisms (EM) biofertilizer, containing key functional microbial groups such as Bacillus, photosynthetic bacteria, yeast, and lactic acid bacteria (effective viable count ≥ 1.0 × 10^8^ CFU g^−1^). It was provided by Hubei Qiming Bioengineering Co., Ltd., and complied with the quality standards of Agricultural Microbial Fertilizers (GB 20287-2006). The bio-organic fertilizer, a mineral-derived humic acid type, contains organic matter ≥40% and N + P₂O₅ + K₂O ≥5.0%, supplied by Henan Liso Crop Protection Co., Ltd. The tested grass species included Qinghai Chinese fescue (*Festuca sinensis* Keng.), Qinghai Kentucky bluegrass (*Poa pratensis* L.), Qinghai alpine bluegrass (*Poa crymophila* Keng.), and Tongde short-awn wheatgrass (*Elymus breviaristatus* Keng f.). These were provided by the Grassland Institute of the Academy of Animal Science and Veterinary Medicine, Qinghai University, with purity ≥92% and germination rate ≥85%.

On May 5, 2022, the tested forage seeds were uniformly mixed and sown in rows at a seeding rate of 18.0 g m^−2^. Fertilization was conducted in early June 2023 and repeated during the same period of 2024 for two consecutive years. Based on preliminary research findings from the team, fertilizer application guidelines, and baseline soil conditions (An et al., 2025; Yu et al., 2025a, 2025b; Zhang L. et al., 2025; Zhang X. L. et al., 2025; Zhang Y. F. et al., 2025), nine treatment groups were established for the combined application of organic fertilizer and EM biofertilizer: organic fertilizer application rates of 1.00 kg m^−2^, 2.00 kg m^−2^, and 3.00 kg m^−2^; EM biofertilizer application rates of 45.00 g m^−2^, 60.00 g m^−2^, and 75.00 g m^−2^ (Table 1). Control group (CK) did not add organic fertilizer and EM bacterial fertilizer. Each experimental plot measures 20 m^2^ (4 m × 5 m) with 2 m inter-plot spacing. Nine fertilization treatments and a CK (control) treatment form one block, with 30 m inter-block spacing. Three replicated blocks were established, totaling 30 experimental plots (see Figure 2c).

**Table 1:** Experimental design for different fertilization treatments.

Treatment group	Organic fertilizer (kg m^−2^)	Effective microorganisms (g m^−2^)
CK	—	—
Y1E1	1.00	45.00
Y1E2	1.00	60.00
Y1E3	1.00	75.00
Y2E1	2.00	45.00
Y2E2	2.00	60.00
Y2E3	2.00	75.00
Y3E1	3.00	45.00
Y3E2	3.00	60.00
Y3E3	3.00	75.00

Y denotes “organic fertilizer,” E denotes “EM biofertilizer,” and numbers 1–3 indicate different application rates (the same applies hereafter).

### 2.3 Vegetation survey and soil sampling

Vegetation height, coverage, density, and biomass were surveyed in September 2023 and the same period in 2024. Five randomly placed 0.5 m × 0.5 m quadrats were established within each experimental plot. Vegetation height was measured using a tape measure (1 mm precision) by averaging five individual plants per grass species. Plant density was calculated by counting all stems within each quadrat (the quadrat area is 0.5 m × 0.5 m). Community coverage was determined using the point-intercept method. Aboveground biomass was quantified by harvesting vegetation at ground level, followed by oven-drying at 105 °C for 30 min and drying at 65 °C for 48 h until constant weight. Plant community height, coverage, density, and biomass for the 10 treatments are presented in Table 2.

**Table 2:** Variations in vegetation height, coverage, density, and biomass under different treatments.

Year	Treatment	Plant height/cm	Plant coverage/%	Density/plant m^−2^	Biomass/(g m^−2^)
2023	CK	16.53 ± 1.60f	58.81 ± 2.47d	345.33 ± 14.47c	198.33 ± 14.19j
	Y1E1	20.04 ± 1.53e	61.80 ± 1.42c	375.67 ± 7.23b	220.00 ± 10.82f
	Y1E2	25.34 ± 0.99d	64.32 ± 1.05bc	398.67 ± 12.42a	261.33 ± 11.15e
	Y1E3	25.96 ± 1.22cd	65.98 ± 1.12b	407.00 ± 7.21a	275.67 ± 11.15de
	Y2E1	25.83 ± 1.36cd	66.33 ± 1.78b	403.67 ± 10.60a	302.00 ± 8.54c
	Y2E2	35.04 ± 1.08a	70.45 ± 1.91a	420.67 ± 9.07a	349.00 ± 10.15a
	Y2E3	30.07 ± 2.78b	65.98 ± 1.13b	410.67 ± 8.50a	323.00 ± 9.17b
	Y3E1	29.09 ± 1.26bc	64.64 ± 1.59bc	404.67 ± 5.51a	326.00 ± 5.57b
	Y3E2	31.01 ± 1.26b	66.16 ± 1.46b	411.33 ± 5.69a	291.33 ± 14.22cd
	Y3E3	29.02 ± 1.38bc	62.91 ± 1.10bc	396.33 ± 9.87a	280.00 ± 7.94de
2024	CK	16.86 ± 1.30e	68.71 ± 2.30e	523.33 ± 14.64e	196.00 ± 5.57e
	Y1E1	18.71 ± 1.25e	75.64 ± 1.51d	560.67 ± 10.26d	222.00 ± 6.00d
	Y1E2	23.75 ± 1.52d	80.96 ± 1.22c	600.00 ± 13.00c	261.67 ± 13.32c
	Y1E3	23.79 ± 1.87d	83.79 ± 1.47b	627.33 ± 12.42b	281.67 ± 6.03c
	Y2E1	27.3 ± 1.43c	85.47 ± 1.59b	632.00 ± 10.15b	320.33 ± 11.37b
	Y2E2	32.59 ± 0.99a	90.66 ± 1.52a	658.33 ± 11.02a	360.67 ± 13.43a
	Y2E3	27.86 ± 2.11c	84.04 ± 1.16b	618.00 ± 17.09bc	331.00 ± 12.29b
	Y3E1	31.09 ± 0.64ab	86.37 ± 0.95b	630.33 ± 9.02b	321.67 ± 9.07b
	Y3E2	32.21 ± 1.10a	81.20 ± 0.75c	622.67 ± 10.97b	281.33 ± 9.29c
	Y3E3	28.99 ± 0.47bc	76.67 ± 1.21d	578.67 ± 11.55d	277.67 ± 7.02c

Data are presented as mean ± standard deviation (SD). Different lowercase letters within the same column indicate significant differences at the 0.05 level (*p* < 0.05).

Soil samples were collected simultaneously, and 0–10 cm soil samples were drilled with 5 cm diameter soil by five-point mixed sampling method. One soil sample is mixed in each plot, which is divided into three parts: one is dry in the shade, which is used to determine the contents of soil organic matter (SOM), total nitrogen (TN) and total phosphorus (TP); One part of wet soil was stored in the refrigerator at −20 °C, and the soil mass moisture content, pH value and conductivity (SEC) were measured. A portion of wet soil was stored in an ultra-low temperature refrigerator at −80 °C for soil microbial diversity analysis.

### 2.4 Measurements and methods

#### 2.4.1 Soil physicochemical properties

Soil electrical conductivity (SEC), soil water content (SWC), pH, soil organic matter (SOM), total nitrogen (TN), and total phosphorus (TP) were determined following the protocols outlined in *Soil Agricultural Chemistry Analysis* (*3rd Edition*) (Gao et al., 2025; Bao, 2005).

#### 2.4.2 Soil bacterial DNA extraction, PCR amplification, and sequencing

Soil samples were sequenced by Shanghai Majorbio Bio-Pharm Technology Co., Ltd. The V3–V4 region of bacterial 16S rDNA was amplified using primers 338F (5′-ACTCCTACGGGAGGCAGCA-3′) and 806R (5′-GGACTACHVGGGTWTCTAAT-3′). The analysis platform was the Illumina MiSeq PE300, with the workflow as follows: soil total DNA extraction, genomic DNA quality control, PCR amplification, agarose gel electrophoresis verification (1%), PCR product purification, MiSeq library construction, MiSeq library quality control, sequencing on the Illumina MiSeq platform. Post-sequencing raw data were processed using the QIIME2 platform (v2020.6). To investigate the species composition of each sample, sequences were aligned against the SILVA database. First, effective tags from all samples underwent OTUs (operational taxonomic units) clustering at 97% identity. Subsequently, taxonomic annotation was performed on the OTUs’ sequences.

### 2.5 Data analysis and visualization

Data organization was conducted using Microsoft Excel 2016. For plant community characteristics and soil physicochemical properties, statistical analysis was performed using IBM SPSS Statistics 22.0 with one-way analysis of variance (ANOVA). Data are presented as mean ± standard deviation (SD), with statistical significance set at *p* < 0.05. Homogeneity of variances was tested using Levene’s test. Visualization analysis of soil bacterial communities (including Venn analysis, abundance analysis, LEfSe statistical analysis, diversity analysis, cluster analysis, and FAPROTAX functional prediction) was conducted through the Majorbio Cloud Platform.^1^ QIIME 2 software was utilized to generate rarefaction curves, relative abundance plots of species, PCoA plots (Bray–Curtis), hierarchical clustering trees, and calculate α-diversity indices including OTUs, Shannon index, Simpson index, Pielou index, Chao1 index, and ACE index. R software (version 3.3.1) was employed to generate alpha diversity boxplots. To investigate differences in community structure among grouped samples, LEfSe statistical analysis was utilized to detect significant differences in species composition and community structure. The FAPROTAX tool was applied to predict and analyze microbial community functions in ecological samples. Network analysis was conducted based on Spearman correlation coefficients (|*r*| > 0.6, *p* < 0.05), and single-factor correlation network diagrams were constructed using NetworkX (version 1.11). R software (version 3.5.2) was used to generate correlation heatmaps between environmental factors, soil physicochemical properties, and microbial diversity. Redundancy analysis (RDA) of environmental factors, soil physicochemical properties, and soil microbial diversity was performed using Canoco 5.0.

## 3 Results

### 3.1 Effects of combined microbial and organic fertilization on soil physicochemical properties

The combined application of microbial biofertilizer and organic fertilizer significantly enhanced soil water content (SWC) and nutrient levels (carbon, nitrogen, phosphorus), while decreasing soil pH and electrical conductivity (SEC) (Table 3). In the second year (2023), the Y2E2 treatment produced a soil water content (SWC) of 26.27%, which was significantly higher than the CK treatment (*p* < 0.05), with an increase of 77.50%. The soil electrical conductivity (SEC) in the Y2E2 treatment was the lowest (818.33 μS cm^−1^), showing a significant reduction of 58.53% compared to CK (*p* < 0.05). The pH value in the Y2E2 treatment decreased to 8.05, significantly lower than that of the CK treatment (*p* < 0.05). The soil organic matter (SOM), total nitrogen (TN), and total phosphorus (TP) contents all reached their peak in the Y2E2 treatment, with values of 253.09 g kg^−1^, 7.50 g kg^−1^, and 2.40 g kg^−1^, respectively. These values represented significant increases of 72.72, 68.92, and 81.82% compared to CK (*p* < 0.05).

**Table 3:** Changes in soil physicochemical properties under different treatments.

Year	Treatment	SWC/%	SEC/(μs cm^−1^)	pH	SOM/(g kg^−1^)	TN/(g kg^−1^)	TP/(g kg^−1^)
2023	CK	14.80 ± 0.82e	1973.33 ± 110.27a	8.78 ± 0.07a	146.53 ± 10.63e	4.44 ± 0.08j	1.32 ± 0.06e
	Y1E1	17.68 ± 1.16d	1614.33 ± 109b	8.62 ± 0.06b	188.32 ± 10.49d	5.05 ± 0.12f	1.61 ± 0.12d
	Y1E2	20.94 ± 1.24c	1333.67 ± 94.88cd	8.50 ± 0.06c	218.40 ± 4.01c	5.58 ± 0.14e	1.82 ± 0.15c
	Y1E3	22.81 ± 1.18bc	1217.00 ± 51.74cd	8.40 ± 0.06cd	226.95 ± 5.27bc	5.97 ± 0.14d	1.99 ± 0.11bc
	Y2E1	24.17 ± 0.37ab	1114.00 ± 76.49d	8.30 ± 0.07de	240.77 ± 3.28ab	6.48 ± 0.23c	2.18 ± 0.12ab
	Y2E2	26.27 ± 1.41a	818.33 ± 111.79e	8.05 ± 0.05f	253.09 ± 10.30a	7.50 ± 0.11a	2.40 ± 0.12a
	Y2E3	24.23 ± 0.40ab	1122.67 ± 140.66d	8.20 ± 0.03e	239.00 ± 7.45ab	6.99 ± 0.16b	2.23 ± 0.06ab
	Y3E1	23.73 ± 1.22abc	1328.67 ± 92.58cd	8.30 ± 0.04de	227.22 ± 8.10bc	6.91 ± 0.09b	2.09 ± 0.11b
	Y3E2	21.47 ± 1.79bc	1401.33 ± 111.85c	8.41 ± 0.04cd	215.79 ± 10.10c	6.78 ± 0.09bc	2.17 ± 0.07ab
	Y3E3	21.68 ± 1.15bc	1448.00 ± 31.95bc	8.38 ± 0.03d	212.69 ± 6.12c	6.71 ± 0.34bc	2.18 ± 0.07ab
2024	CK	13.83 ± 0.61e	2086.67 ± 103.02a	8.82 ± 0.06a	130.33 ± 7.52e	4.39 ± 0.12j	1.27 ± 0.06e
	Y1E1	18.49 ± 1.07d	1559.00 ± 132.55b	8.56 ± 0.11b	200.68 ± 4.97d	5.47 ± 0.07f	1.67 ± 0.07d
	Y1E2	22.96 ± 1.30c	1175.33 ± 103.36c	8.40 ± 0.10c	226.40 ± 9.57c	5.86 ± 0.06e	1.90 ± 0.13c
	Y1E3	23.86 ± 0.43bc	1174.67 ± 116.80c	8.30 ± 0.08cd	239.48 ± 11.93abc	6.38 ± 0.18d	2.11 ± 0.06b
	Y2E1	26.05 ± 0.58ab	1042.67 ± 51.50c	8.20 ± 0.08de	258.98 ± 16.85ab	6.76 ± 0.11c	2.32 ± 0.05a
	Y2E2	28.12 ± 1.23a	714.00 ± 99.45d	7.84 ± 0.08f	261.44 ± 8.77a	7.74 ± 0.09a	2.45 ± 0.05a
	Y2E3	26.1 ± 1.15ab	1065.33 ± 34.95c	8.11 ± 0.07e	249.03 ± 12.62abc	7.24 ± 0.17b	2.39 ± 0.07a
	Y3E1	25.28 ± 1.47abc	1175.67 ± 96.2c	8.17 ± 0.05de	240.52 ± 15.28abc	7.15 ± 0.16b	2.41 ± 0.14a
	Y3E2	27.75 ± 1.22a	1286.33 ± 109.62c	8.36 ± 0.11cd	233.65 ± 9.59abc	7.00 ± 0.10bc	2.38 ± 0.07a
	Y3E3	26.64 ± 2.47ab	1269.33 ± 58.16c	8.36 ± 0.05cd	230.68 ± 9.27bc	6.88 ± 0.09c	2.43 ± 0.09a

Data are presented as mean ± standard deviation (SD). Different lowercase letters within the same column indicate significant differences at the 0.05 level (*p* < 0.05). SWC, soil water content; SEC, soil electrical conductivity; SOM, soil organic matter; TN, soil total nitrogen; TP, soil total phosphorus.

In the third year (2024), the trends in soil property changes remained consistent with the second year, and the improvement effects were even more pronounced. In the Y2E2 treatment, soil water content (SWC) further increased to 28.12%, and soil electrical conductivity (SEC) decreased to 714.00 μS cm^−1^, both showing significant differences compared to the CK treatment (*p* < 0.05). The pH value significantly decreased to 7.84, further approaching neutral conditions. The soil organic matter (SOM), total nitrogen (TN), and total phosphorus (TP) contents in the Y2E2 treatment reached 261.44 g kg^−1^, 7.74 g kg^−1^, and 2.45 g kg^−1^, respectively, representing significant increases of 100.60, 76.31, and 92.91% compared to the CK treatment (*p* < 0.05).

### 3.2 Effects of combined microbial and organic fertilization on soil bacterial community

#### 3.2.1 Quality analysis of soil bacterial 16S sequencing results and changes in OTU counts

As shown in Supplementary Figure S1a, when the sequence extraction number reached 5,000, the rarefaction curves of different treatment groups plateaued as the sample size increased (30,000 reads sparse threshold). This indicated that the current sequencing data volume approached saturation, and the sequencing depth was appropriately set. Further increasing the data volume would only identify a minimal number of low-abundance species. Venn analysis (Supplementary Figure S1b) revealed that a total of 2,549 bacterial OTUs (operational taxonomic units) were detected across the 10 treatment groups. Among these, the Y2E3 and Y2E2 treatments had the highest OTU counts, with 2,501 and 2,481 OTUs, respectively, while the CK treatment had 2,459 OTUs. A total of 2,042 OTUs were shared among all 10 treatment groups, accounting for 80.11% of the total OTUs. This indicates a high similarity in bacterial community composition across different treatment groups, with a limited distribution of unique OTUs.

#### 3.2.2 Composition and relative abundance changes of soil bacterial communities

**Figure 3:**
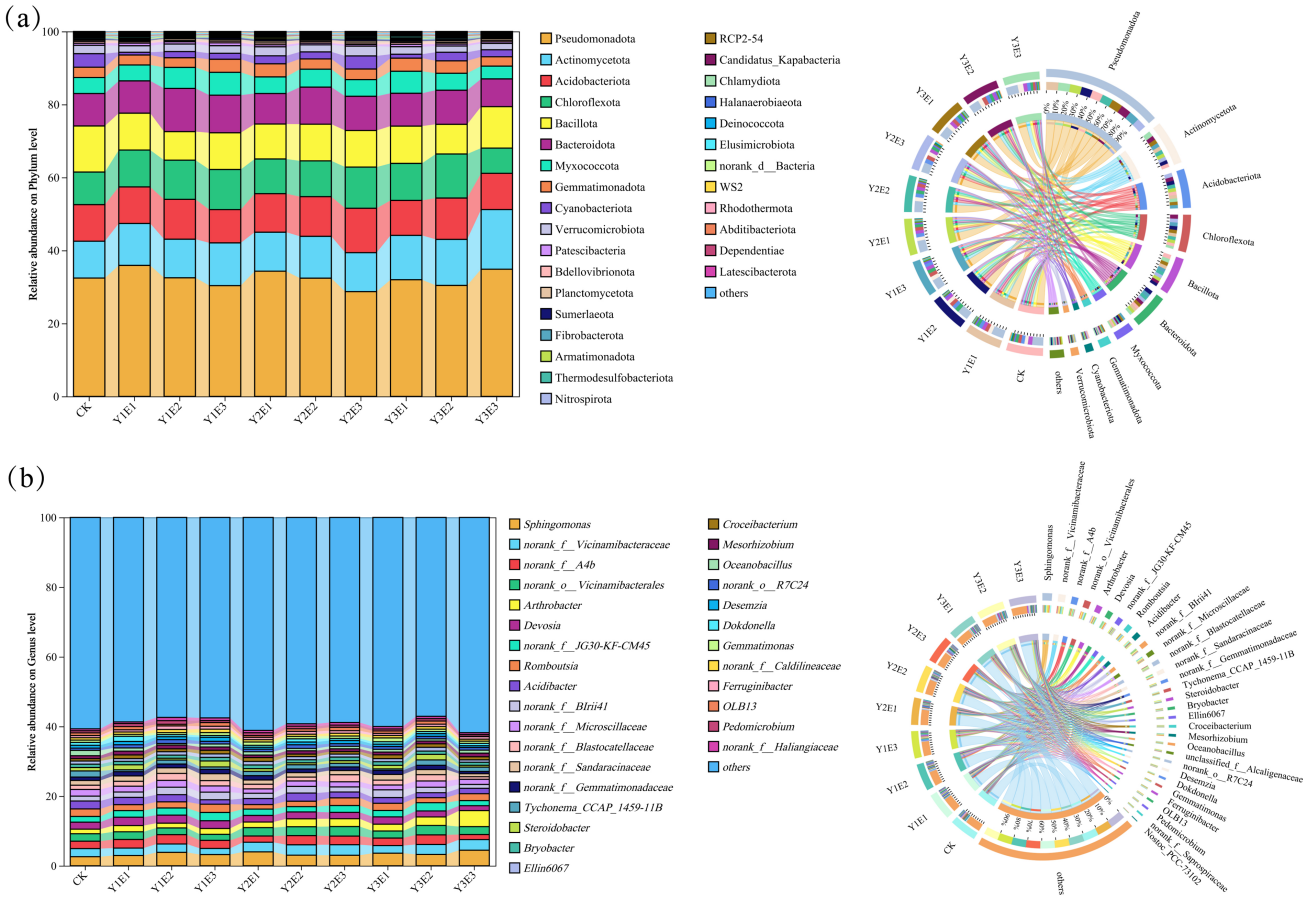
**(a)** Changes in the relative abundance of soil bacterial phyla across different treatments. **(b)** Changes in the relative abundance of soil bacterial genera across different treatments.

The relative abundances of the top 30 soil bacterial phyla under different fertilization treatments are shown in Figure 3a. Dominant phyla included Pseudomonadota (28.71–35.91%), Actinomycetota (0.09–16.35%), Acidobacteriota (9.11–12.16%), Chloroflexi (6.91–12.07%), Bacillota (7.82–12.63%), and Bacteroidota (7.60–11.85%), which consistently ranked among the highest in relative abundance across all 10 treatments. Significant differences in the relative abundances of soil bacterial phyla were observed between the CK treatment and the nine fertilization treatments. Compared with CK, the Y2E2 and Y2E3 treatments increased the abundances of Actinomycetota and Acidobacteriota while reducing the abundance of Pseudomonadota.

At the genus level (Figure 3b), *Sphingomonas* (2.61–4.48%), *norank_*f__Vicinamibacteraceae (1.76–33.04%), *norank*_f__A4b (1.46–2.76%), *norank_*o__Vicinamibacterales (1.73–2.74%), *Arthrobacter* (1.32–4.60%), and *Devosia* (1.42–2.42%) exhibited relatively high abundances. Further analysis revealed that, compared to the CK treatment, the Y2E2 and Y2E3 treatments increased the abundance of *norank_*f__Vicinamibacteraceae while reducing the abundance of *Devosia*.

#### 3.2.3 LEfSe analysis of soil bacterial communities

As shown in Supplementary Figure S2, LEfSe analysis identified the most significant indicator species contributing to bacterial community differences across the 10 treatments (LDA score >3.0). A total of 98 biomarkers were detected in the bacterial communities of the 10 treatments (8 in Y2E2 and 10 in each of the other treatments). At the taxonomic level, the highest-scoring biomarkers for the CK, Y1E1, Y1E2, Y1E3, Y2E1, Y2E2, Y2E3, Y3E1, Y3E2, and Y3E3 treatments were f__norank_o*__Chloroplast*, f*__Devosiaceae*, c*__Bacteroidia*, c_*_Polyangiia*, g*__Castellaniella*, g*__*norank*_f__Rhodanobacteraceae*, f*__Nostocaceae*, c*__Gemmatimonadia*, p*__Chloroflexota*, and g*__Arthrobacter*, respectively.

#### 3.2.4 Analysis of soil bacterial community diversity

**Figure 4:**
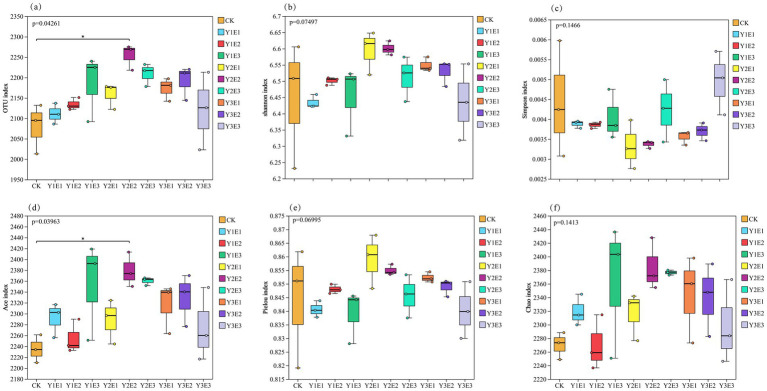
Differences in soil bacterial community α-diversity across different treatments. ^*^Represents *p* < 0.05. **(a)** OTUs index, **(b)** Shannon index, **(c)** Simpson index, **(d)** Ace index, **(e)** Pielou index, **(f)** Chao1 index.

As shown in Figure 4, significant differences were observed in the α-diversity indices of soil bacterial communities across the 10 treatments. The Y2E2 treatment exhibited the highest values for OTU number, Shannon index, Ace index, and Chao1 index, with values of 2,254, 6.60, 2,379.10, and 2,384.80, respectively. These values represented increases of 8.37, 2.36, 6.44, and 5.05% compared to the CK treatment, with the OTU number and Ace index reaching significant levels (*p* < 0.05). The Pielou index reached its maximum value in the Y1E2 treatment (0.86), followed by the Y2E2 treatment (0.85), representing increases of 1.77 and 1.30% compared to CK. Among the 10 treatments, the Simpson index values ranked from highest to lowest as follows: Y3E3 > CK > Y2E3 > Y1E3 > Y1E1 > Y1E2 > Y3E2 > Y3E1 > Y2E2 > Y2E1, with the Y2E2 and Y2E1 treatments exhibiting the lowest Simpson index values. In summary, the combined application of EM microbial biofertilizer and organic fertilizer significantly enhanced the α-diversity of soil bacterial communities, with the Y2E2 treatment performing the best, followed by the Y2E1 treatment.

**Figure 5:**
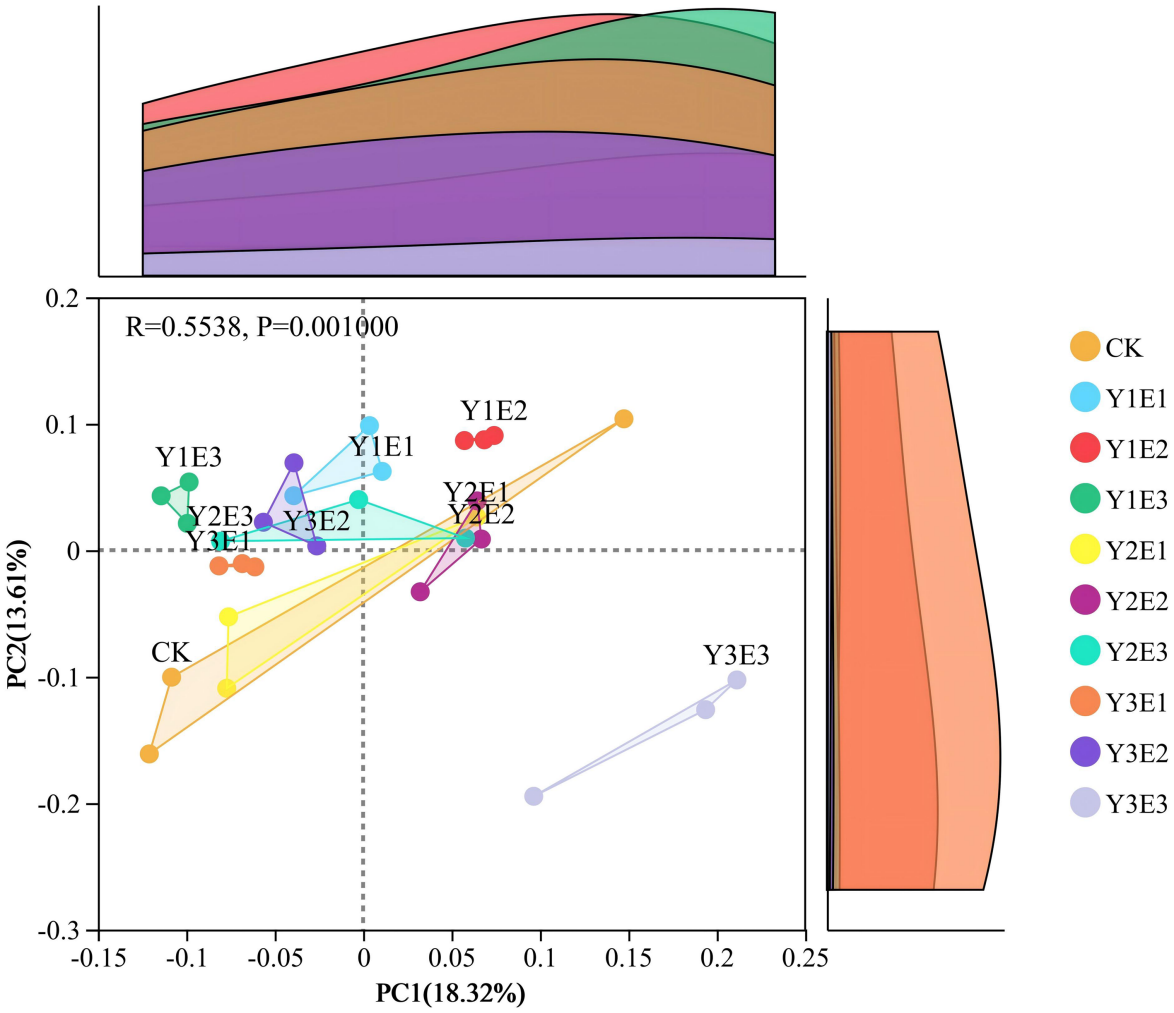
Differences in soil bacterial community β-diversity (PCoA) across different treatments.

Principal coordinate analysis (PCoA) based on Bray–Curtis distance was performed for the 10 treatments, and the results were shown in Figure 5. In the CK treatment, soil bacterial communities exhibited low aggregation and high within-group variation. In contrast, the nine fertilization treatments showed higher aggregation and lower within-group variation, with the Y1E2 and Y3E1 treatments displaying the highest aggregation and the smallest within-group variation. The first principal coordinate (PC1) explained 18.32% of the variance, while the second principal coordinate (PC2) explained 13.61%, with a cumulative explanation rate of 31.93%. Significant differences were observed between groups (*p* = 0.001), and the communities were distinctly clustered into three groups: Y3E3 formed a separate group, the remaining eight fertilization treatments clustered into one group, and the CK treatment formed another group.

#### 3.2.5 Changes in soil bacterial community single-factor correlation networks

**Figure 6:**
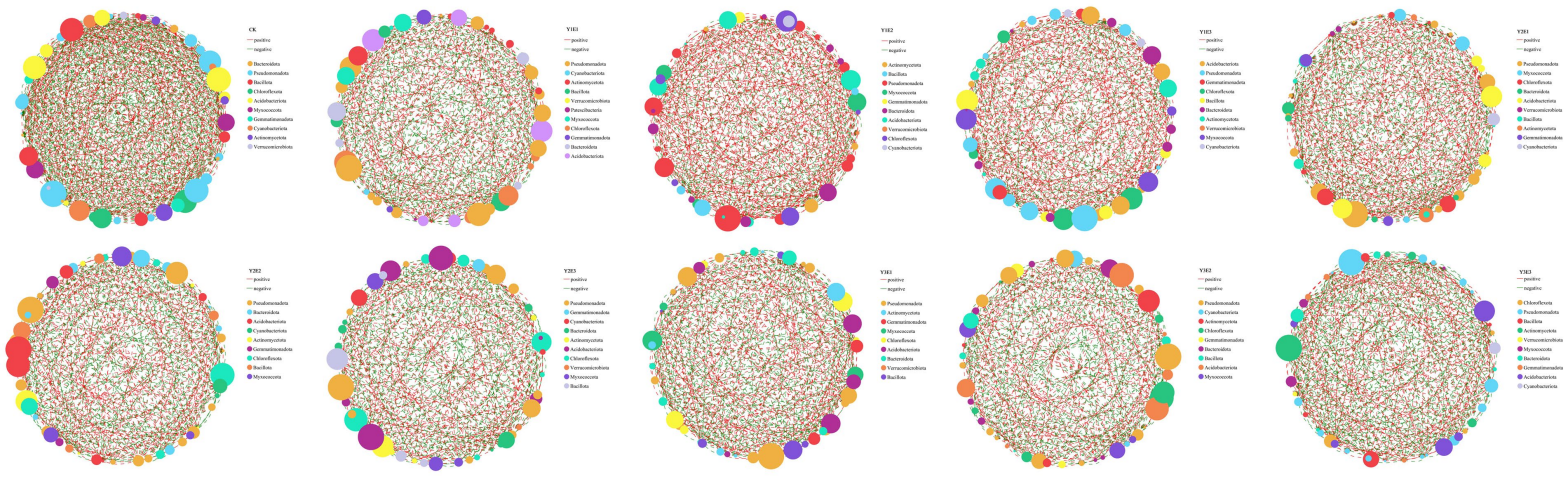
Single-factor correlation networks of soil bacteria across different treatments. Node color and size indicate species type and importance; line color indicates positive or negative correlation, with red positive and green negative; and the number of lines indicates whether the species are closely related.

To explore the co-occurrence characteristics and interaction relationships of bacterial communities at the genus level under different fertilization treatments, single-factor correlation networks were constructed (Figure 6). The results revealed significant differences in the structural characteristics of soil bacterial networks among treatments. The number of edges in the bacterial networks ranged from 376 to 656, showing significant variation, while the number of nodes exhibited smaller differences among treatments. The proportion of positive correlations in the bacterial networks varied significantly across treatments, with the Y1E2 treatment displayed the highest positive correlation (67.11%), followed by Y1E3 (64.52%). In contrast, the CK, Y1E1, Y2E3, Y3E2, and Y3E3 treatments had lower positive correlations, all below 50.0%, with values of 49.39, 48.26, 48.74, 48.14, and 48.58%, respectively. The key connecting nodes in the single-factor correlation networks also differed significantly among treatments. In the nine fertilization treatments, Pseudomonadota, Actinomycetota, Cyanobacteriota, and Bacillota were the most important connecting nodes. In the control treatment (CK), Bacteroidota, Pseudomonadota, Bacillota, and Chloroflexota served as key connecting nodes.

#### 3.2.6 Functional prediction of soil bacterial communities

**Figure 7:**
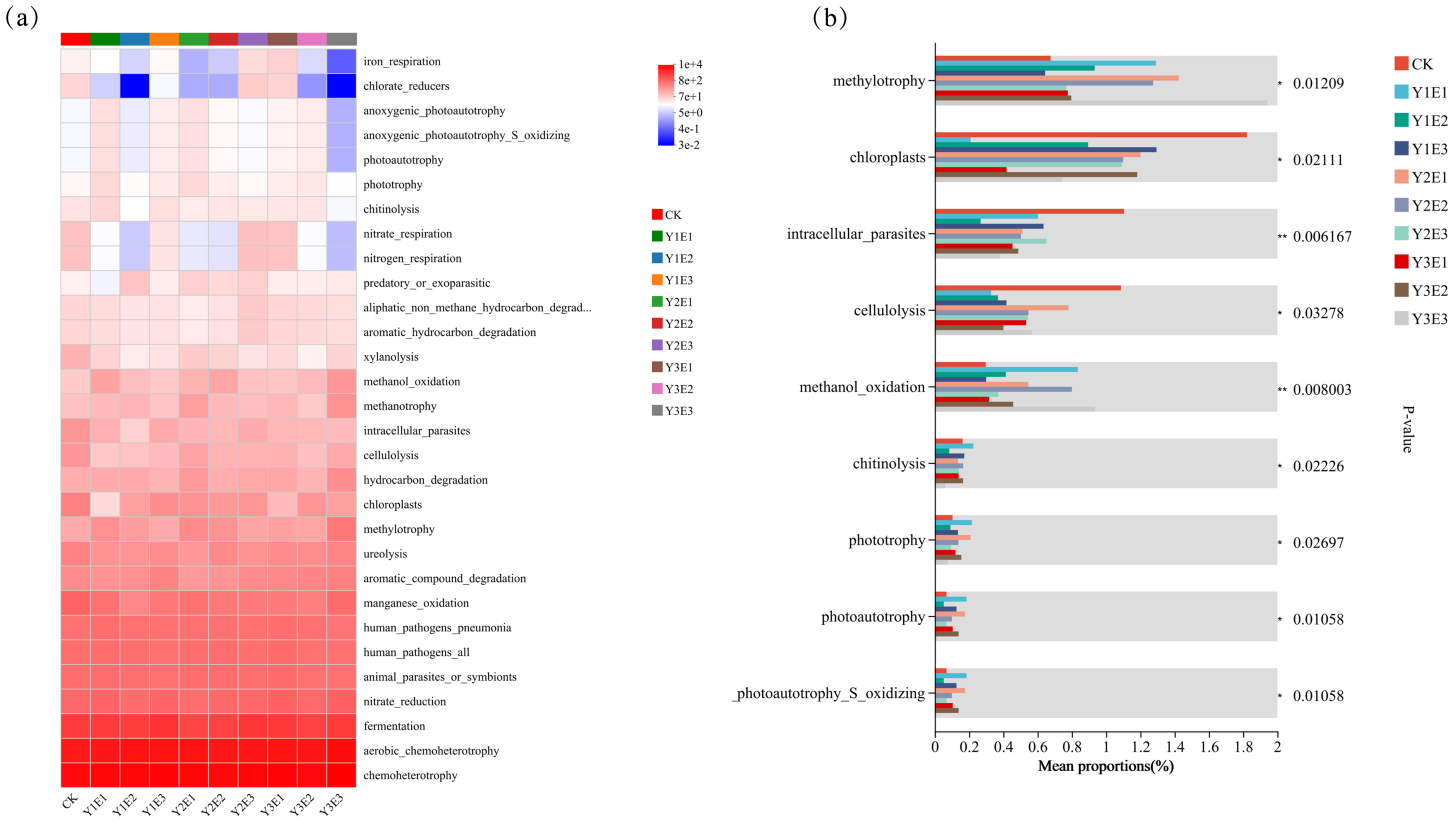
Prediction of FAPROTAX function of soil bacteria in different treatments: heatmap and inter-group test analysis. **(a)** Prediction of FAPROTAX functions of soil bacteria, **(b)** Inter-group test analysis of FAPROTAX functional groups of soil bacteria.

Functional annotation of bacterial communities across the 10 treatments was performed using the FAPROTAX tool, identifying 30 sub-functions. As shown in Figure 7 fermentation, aerobic chemoheterotrophy, and chemoheterotrophy were the dominant functions across all treatments. Specifically, chemoheterotrophy was most pronounced in Y3E3 (10,253), Y3E1 (9,266), and Y1E3 (9,284); aerobic chemoheterotrophy was highest in Y3E3 (7,571), Y3E1 (6,616), and Y2E1 (6,501); and fermentation was most intense in Y1E3 (2,888), Y2E3 (2,696), and Y3E1 (2,526). Additionally, the manganese oxidation function was significantly higher in Y1E1, Y2E2, and Y3E3 compared to CK. Similarly, chloroplast function was significantly elevated in Y1E3, Y2E3, and Y3E2 compared to CK.

Further analysis revealed significant differences in specific metabolic functions among treatments. Chlorate-reducers function was significantly lower in Y1E2 (0.03) and Y3E3 (0.033) compared to CK. In Y3E3, iron respiration, anoxygenic photoautotrophy, anoxygenic photoautotrophy_S_oxidizing, and photoautotrophy functions were all lower than in CK. Nitrate respiration and nitrogen respiration functions were significantly lower in Y1E2, Y2E1, Y2E3, and Y3E3 compared to CK. Conversely, anoxygenic photoautotrophy, anoxygenic photoautotrophy_S_oxidizing, and photoautotrophy abundance were significantly lower in CK compared to other fertilization treatments.

#### 3.2.7 Clustering analysis of soil bacterial communities

Hierarchical clustering analysis was performed on 30 soil samples based on distance metrics. As shown in Supplementary Figure S3, the bacterial communities across the 10 treatments were divided into two groups: Y3E3 formed a separate group (Group 1), while the CK treatment and other fertilization treatments were grouped together (Group 2). Further analysis revealed that, at a clustering threshold of 0.17, Group 2 could be subdivided into two subgroups: CK treatment formed a distinct subgroup, while the remaining fertilization treatments clustered into another subgroup. Additionally, the three soil samples from Y1E2, Y2E2, Y1E1, Y3E2, and Y1E3 treatments were located on the same branch, indicating high similarity in their soil bacterial community structures. In contrast, the CK treatment and other fertilization treatments were distributed on different branches, reflecting differences in their soil bacterial community composition.

### 3.3 Coupling relationships among vegetation community characteristics, soil physicochemical properties, and soil bacterial communities

#### 3.3.1 Mantel test analysis of vegetation community characteristics, soil physicochemical properties, and soil bacterial communities

**Figure 8:**
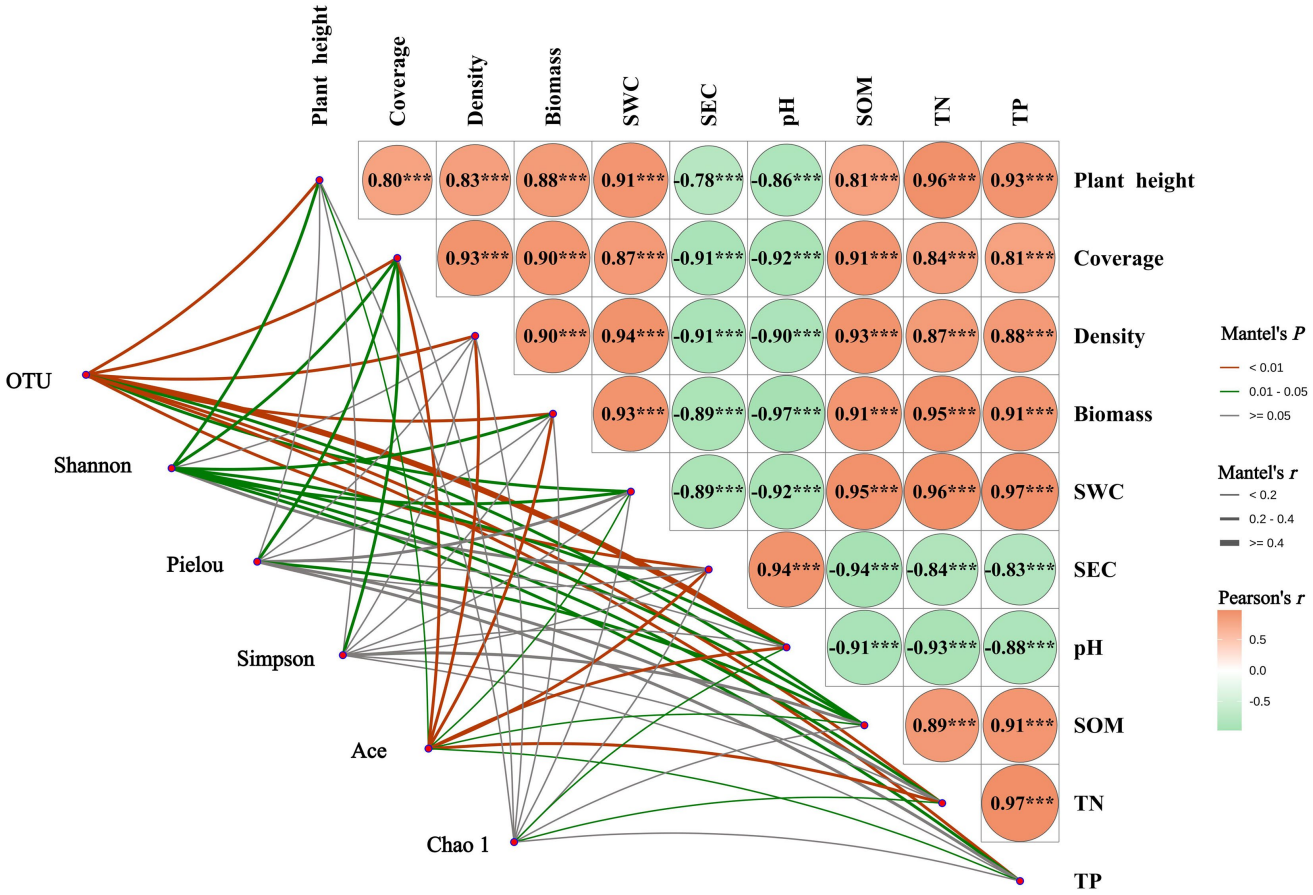
Mantel analysis of vegetation community characteristics, soil physicochemical properties, and soil bacterial communities. ^***^Denotes *p* < 0.001; SWC, soil water content; SEC, soil electrical conductivity; SOM, soil organic matter; TN, soil total nitrogen; TP, soil total phosphorus.

As shown in Figure 8, the Mantel test revealed a highly significant positive correlation (*p* < 0.001) between soil pH and electrical conductivity (SEC), while both exhibited highly significant negative correlations (*p* < 0.001) with other soil physicochemical properties and vegetation characteristics. Plant height, coverage, density, and aboveground biomass were all significantly positively correlated (*p* < 0.001) with soil water content (SWC), organic matter (SOM), total nitrogen (TN), and total phosphorus (TP). The number of bacterial OTUs showed significant correlations (*p* < 0.05) with SOM and SWC, while exhibiting highly significant correlations (*p* < 0.05) with other soil physicochemical properties and vegetation characteristics. The Shannon index was significantly correlated (*p* < 0.05) with all soil physicochemical properties and vegetation characteristics except density and SEC. The Pielou index showed significant correlations (*p* < 0.05) only with coverage and SOM. The Simpson index was significantly correlated (*p* < 0.05) only with coverage. The Ace index exhibited significant (*p* < 0.05) or highly significant (*p* < 0.01) correlations with all four vegetation characteristics and six soil physicochemical properties. The Chao1 index showed significant correlations (*p* < 0.05) only with pH and TN.

#### 3.3.2 Redundancy analysis of vegetation community characteristics, soil physicochemical properties, and soil bacterial communities

**Figure 9:**
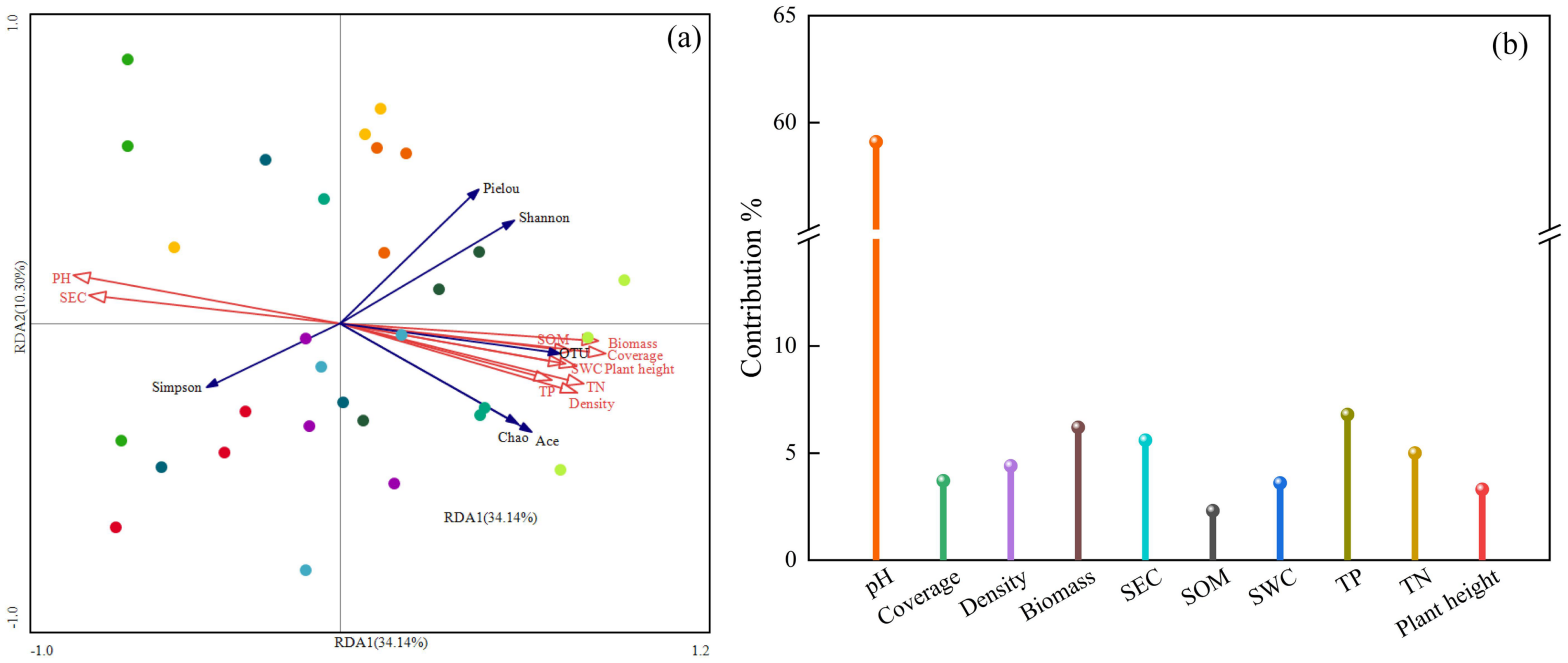
RDA analysis of soil physicochemical properties, plant community characteristics, and soil bacterial community structure. Red arrows indicate plant community characteristics and soil factors, and blue arrows indicate bacterial diversity indices. SWC, soil water content; SEC, soil electrical conductivity; SOM, soil organic matter; TN, soil total nitrogen; TP, soil total phosphorus. **(a)** RDA analysis, **(b)** Contribution rates of plant and soil factors.

Redundancy analysis (RDA) was conducted to explore the relationships between soil bacterial community diversity, vegetation characteristics, and soil physicochemical factors, as shown in Figure 9. The first (RDA I) and second (RDA II) axes explained 34.14 and 10.30% of the variance, respectively, with a cumulative explanation rate of 44.44%. Among the factors, soil pH had the longest arrow length, explaining 27.5% of the variance and contributing 59.1% to the model, with a significant effect (*p* = 0.002). This indicates that soil pH is the most significant factor influencing bacterial community diversity in the mining area. Soil pH and electrical conductivity (SEC) were positively correlated with the Simpson index but negatively correlated with the Shannon index, OTU number, Ace index, and Chao1 index. Other soil physicochemical factors [e.g., organic matter (SOM), total nitrogen (TN), total phosphorus (TP)] and vegetation characteristics were positively correlated with bacterial diversity indices (except the Simpson index).

## 4 Discussion

### 4.1 Effects of EM bacterial fertilizer combined with organic fertilizer on soil physicochemical properties and community characteristics

The application of microbial fertilizers introduces exogenous functional microbial communities into the soil, thereby revitalizing the soil ecosystem. When further combined with organic fertilizer application, it enhances organic matter decomposition processes, facilitates the conversion of organic nutrients into readily available forms, and thus improves soil nutrient cycling efficiency (Zhao et al., 2021; Pereg and McMillan, 2015). Multiple studies have demonstrated that the combined application of microbial fertilizers with organic/chemical fertilizers significantly increases vegetation biomass in degraded grassland restoration (Gao et al., 2025; Ren et al., 2021; XU et al., 2025; Ramakrishna et al., 2019). This study found that the combined application of EM bacterial fertilizer and organic fertilizer (Y2E2 treatment) consistently promoted vegetation growth during two consecutive growing seasons, with biomass increasing by 75.97 and 84.02% compared to the control (CK). Concurrently, soil water content (SWC), soil organic matter (SOM), and total nitrogen (TN) content were significantly enhanced. However, a distinct threshold effect was observed in application dosage—excessive application inhibited vegetation growth, which aligns with the findings of Song et al. (2015) and Cai et al. (2018).

Soil nutrients, as the material basis for plant growth, directly govern vegetation development through their effective availability (Jyoti et al., 2024). Data from this study reveal that the Y2E2 treatment synergistically enhanced the physicochemical properties of the 0–10 cm topsoil layer via the combined application of microbial fertilizer and organic fertilizer. Over 2 years, this treatment significantly increased soil water content (SWC), soil organic carbon (SOC), total nitrogen (TN), and total phosphorus (TP) compared to the control, while markedly reducing soil electrical conductivity (SEC) and pH levels. This improvement effect stems from functional bacterial strains enhancing nitrogen and phosphorus availability by accelerating the mineralization process of organic fertilizer (Chen et al., 2025). Additionally, organic acids produced by soil bacterial metabolism promote organic matter decomposition and neutralize alkaline substances through proton release, creating a microenvironment conducive to nutrient activation (Simon et al., 2024). These findings align with studies by Song et al. (2019) and Ku et al. (2018), which confirmed that co-application of microbial and organic fertilizers enhances soil nitrogen reserves and organic matter accumulation. Notably, a threshold effect governs the combined application: excessive use (e.g., Y3E3 treatment) elevates risks of nutrient leaching, reduces nutrient turnover efficiency, and triggers salinization and pH instability (Kong et al., 2013). In this study, Y3E3 exhibited slightly higher pH and SEC than Y2E2, indicating that soil buffering capacity becomes compromised under excessive nutrient inputs (Wang S. et al., 2025; Wang Z. et al., 2025). Chu (2014) further observed that surpassing critical application rates increases soil conductivity, corroborating the physicochemical degradation observed here due to over-application. Therefore, precise regulation of the microbial organic fertilizer ratio is critical for optimizing nutrient conversion efficiency and preserving soil ecological stability (Yang et al., 2024; Xu, 2011; Zhong et al., 2020).

### 4.2 Effects of EM bacterial fertilizer combined with organic fertilizer on soil bacterial community structure and function

Among the 10 treatments in the Muli mining area, Pseudomonadota and Actinomycetota were the two dominant bacterial phyla with the highest relative abundances in the soil, indicating their critical functional roles in the bacterial communities of this artificial grassland (Yang et al., 2023; Xu, 2011; Zhong et al., 2020). Numerous studies have demonstrated that variations in soil nutrient content significantly reshape bacterial community composition (Yang et al., 2023; Lian et al., 2025; Xu et al., 2021). In the Y2E3 treatment group, Actinomycetota and Acidobacteriota exhibited a synergistic enrichment pattern, with their abundances significantly surpassing those in the CK treatment. The primary reason lies in the functional complementarity of soil bacteria: Actinomycetota bacteria drive soil carbon cycling by decomposing lignin and other complex organic matter, while Acidobacteriota bacteria stabilize the soil carbon reservoir. This functional complementarity between Actinomycetota and Acidobacteriota within soil bacteria enhances carbon conversion efficiency in soils (Lian et al., 2025). Xu et al. (2021) observed that habitats with high phosphorus content and healthy vegetation communities harbor higher relative abundances of Sphingomonas (a genus within Pseudomonadota) and Arthrobacter (a genus within Actinomycetota). This correlation likely stems from their dual nutritional traits: plant growth promotion capabilities; Optimization of soil phosphorus transformation, with their abundances positively correlated with soil phosphorus levels. Consistent with these findings, the current study revealed significantly elevated relative abundances of Sphingomonas and Arthrobacter in fertilized treatments compared to CK, further validating their role in phosphorus cycling and ecosystem health.

Soil bacteria, as the core components regulating soil ecosystem functions, play a pivotal role in governing material cycling and energy flow within plant–soil systems through their community structure and functional traits (Yang et al., 2024). This study found that the combined application of EM bacterial fertilizer and organic fertilizer significantly influenced soil bacterial community diversity. In the Y2E2 treatment group, compared to the control treatment (CK), the number of OTUs (operational taxonomic units) and the Ace index increased by 8.37 and 6.44%, respectively. These findings align with the conclusions of Xu (2011) regarding the combined application of microbial and organic fertilizers. The mechanism is that organic fertilizer provides complex carbon sources, while nitrogen-fixing bacteria and phosphate-solubilizing bacteria in EM microbial fertilizer accelerate organic matter decomposition by secreting extracellular enzymes. These effects create diversified soil niches, thereby enhancing the diversity of bacterial communities in artificially restored grassland soils (Zhong et al., 2020). Further single-factor correlation network analysis revealed that the rhizosphere bacterial network complexity in the Y1E2 treatment group was significantly enhanced compared to CK, with a positive correlation ratio of 67.11%. This indicates that most bacterial populations in this treatment formed a tightly collaborative network through strategies such as mutualistic symbiosis (e.g., nutrient exchange) and cooperative commensalism (e.g., metabolic complementarity). Such a highly connected bacterial structure optimizes rhizosphere nutrient turnover efficiency and accelerates the synchronized metabolic activities of nitrogen-fixing and phosphorus-solubilizing bacteria (Che et al., 2024; Zhang et al., 2025; Zhang et al., 2025; Zhang et al., 2025).

Li et al. (2025) demonstrated that alterations in soil bacterial community structure led to distinct functional characteristics. This study corroborates their findings through FAPROTAX-based predictions of bacterial functions across different fertilization treatments, revealing significant functional divergence among the 10 treatments. Notably, the Y1E1 and Y2E2 treatments exhibited markedly higher manganese oxidation functional intensity compared to other groups. This enhancement likely stemmed from the abundant photosynthetic bacteria in EM fertilizer, which establish a unique photoenergy-manganese oxidation coupled metabolic pathway via light-driven CO₂ fixation (Wang S. S. et al., 2025; Wang Z. X. et al., 2025; Cai et al., 2025; Cohen et al., 2022). The concurrent significant increase in chloroplast-related functional intensity in these treatments further supports this hypothesis. Critically, the Y3E3 treatment group showed suppressed functional intensities in chlorate reduction, iron respiration, and phototrophic autotrophy, underscoring that excessive combined application may inhibit specific bacterial metabolic functions, reinforcing the existence of a threshold effect (Zhong et al., 2020; Yang et al., 2023). The functional intensity of photosynthetic autotrophic-related functions in soil bacteria under the control treatment (CK) was significantly lower than that under other fertilization treatments. The potential reason may be attributed to the chronic deficiency of organic matter input in the *in situ* soil of the Muli mining area (Qinghai Province, China), which has resulted in a severe shortage of substrates (e.g., sulfides, low-molecular-weight organic compounds) required for photosynthetic bacteria growth, thereby constraining their metabolic activity.

### 4.3 Correlation analysis of rhizosphere soil microbial composition with soil physicochemical properties and plant community characteristics

Mantel test analysis revealed that soil pH exhibited an extremely significant positive correlation with SEC (*p* < 0.001), while both variables demonstrated extremely significant negative correlations with other soil physicochemical properties and vegetation community characteristics (*p* < 0.001). These findings align with research by Gao et al. (2025), which posits that higher soil nutrient and moisture levels promote superior plant community growth. Redundancy analysis (RDA) further demonstrated that soil SEC was negatively correlated with bacterial community diversity indices (Shannon, Ace, and Chao1), indicating that soil salinity stress significantly inhibits the recovery of bacterial diversity (Luo et al., 2025). In contrast, vegetation community traits and soil fertility indicators soil organic matter (SOM), total nitrogen (TN), and total phosphorus (TP) exhibited significant positive correlations with bacterial diversity indices (except the Simpson index). This suggests that organic matter and mineral nutrients (N, P) enhance bacterial community structural succession and functional differentiation by supplying energy and metabolic substrates, thereby fostering diversity restoration. In summary, the vegetation restoration process in the Muli mining area of Qinghai achieves bidirectional enhancement of soil fertility improvement and bacterial community optimization through the “vegetation-soil-microbe” interaction mechanism, ultimately establishing a self-reinforcing ecological virtuous cycle.

Soil physicochemical properties exert a significant shaping effect on the diversity and structure of soil bacterial communities in artificial grasslands within the Muli mining area of Qinghai Province. Unlike traditional studies that prioritize soil nutrient availability as the core driver of bacterial community distribution, this study highlights the dominant regulatory role of pH (Liu et al., 2018). This finding is highly consistent with the “rugby ball model” proposed by Xun et al. (2015). The model posits that when soil pH falls within the neutral range (6.5–7.5), soil nutrient conditions primarily govern changes in bacterial community structure and functional dynamics. However, when pH shifts to acidified (5.5–6.5) or alkalized (7.5–8.5) ranges, pH value becomes the primary controlling factor influencing bacterial community structure and functional dynamics. In the study area, soil pH ranged from 7.84 to 8.82 (characteristic alkaline conditions), consistent with their model. Thus, pH not only influences rhizosphere microbial communities in grasslands through soil fertility but also regulates bacterial community stability via plant-microbe interaction networks. For ecological restoration practices, adjusting soil pH to the neutral range (6.5–7.5) can maximize bacteria-driven ecological services such as carbon cycling and nutrient transformation, optimizing ecosystem functionality.

## 5 Conclusion

1. The combined microbial fertilizer treatment demonstrated significant improvement on soil properties in the Muli mining area. Notably, the Y2E2 treatment exhibited remarkable enhancement in soil nutrient indicators, with soil organic matter content reaching 253.09 g kg^−1^, representing a 72.72% significant increase compared to the CK treatment. Additionally, soil electrical conductivity and pH values under Y2E2 were significantly reduced relative to CK.
2. The Y2E2 treatment elevated soil bacterial diversity indices, increasing the Shannon, Ace, and Chao1 indices by 2.36, 6.44, and 5.05%, respectively, compared to CK. The Ace index improvement reached statistical significance (*p* < 0.05).
3. The Y1E2 treatment showed a 67.11% positive correlation ratio in single-factor correlation networks, significantly higher than CK and other microbial fertilizer treatments. Core functional microbial phyla included Pseudomonadota, Actinobacteria, and Acidobacteria.
4. Hierarchical clustering analysis divided soil bacterial communities across 10 treatments into two distinct groups: Y3E3 formed an independent cluster, CK constituted a separate group, while other fertilization treatments aggregated into another cluster. Functional prediction revealed that all fertilization treatments except CK significantly enhanced anaerobic photoautotrophy, sulfur-oxidizing anaerobic photoautotrophy, and photoautotrophic functional capacities.
5. Redundancy analysis (RDA) revealed that soil pH was the core environmental factor of soil bacterial community succession in Muli mining area, Qinghai Province (*p* = 0.002), which not only directly promoted bacterial diversity, but also indirectly affected bacterial metabolic function by regulating soil nutrient content and vegetation community.
6. Comprehensive analysis identified Y2E2 as the optimal treatment, achieved through combined application of 600.00 kg EM biofertilizer and 20.00 t organic fertilizer per hectare. This regimen effectively coordinated the structural and functional restoration of the alpine mining grassland ecosystem.

## Data Availability

The data supporting the findings of this study are available in the NCBI repository under accession number PRJNA1298863.

## Footnotes

^1^ https://www.majorbio.com
