# Supplementary material for: EM biofertilizer and organic fertilizer co-application modulate vegetation-soil-bacteria interaction networks in artificial grasslands of alpine mining regions
Source: Front Microbiol. 2025 Sep 18;16:1659475. doi: 10.3389/fmicb.2025.1659475 (PMC12488561; doi:10.3389/fmicb.2025.1659475)
Supplement: Supplementary file 1 [file Data_Sheet_1.docx]

Supplementary Material

# Supplementary Figures

**
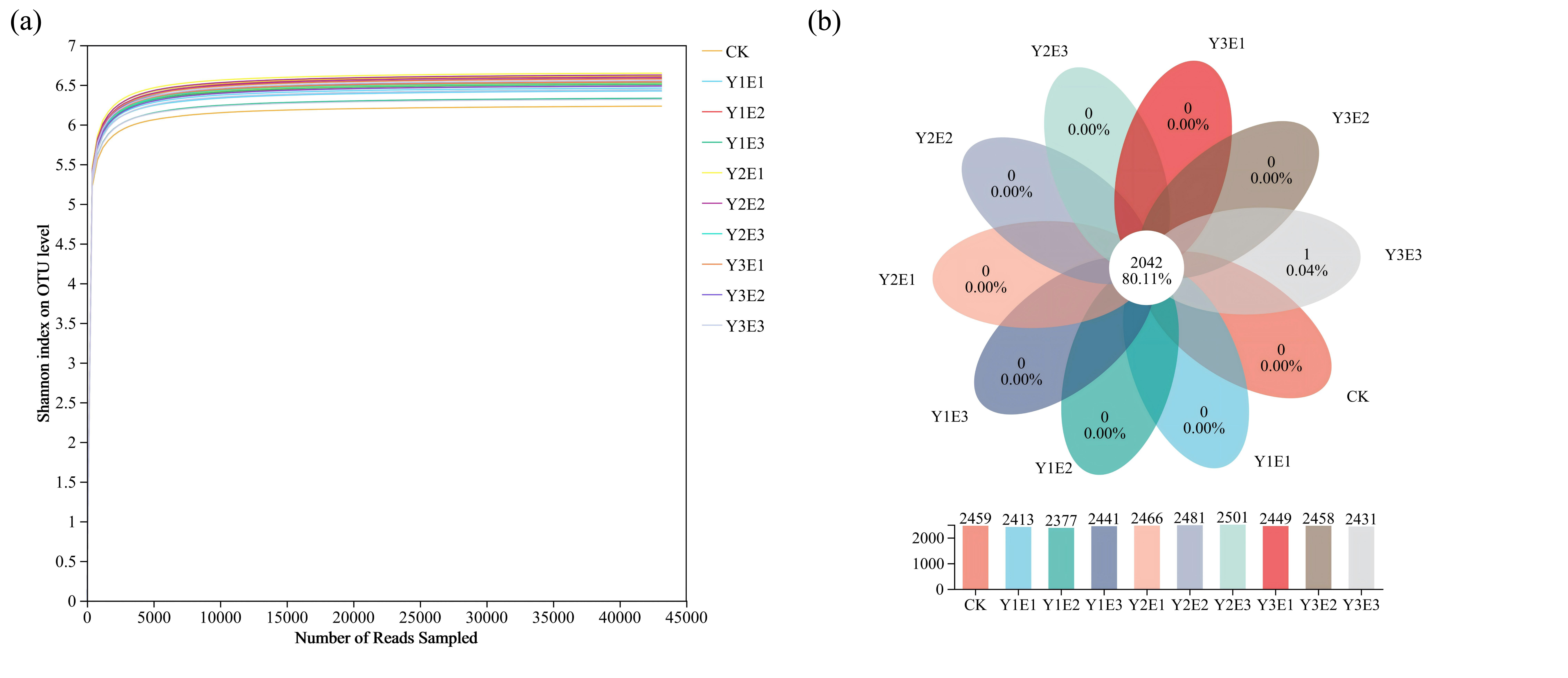
**

**Supplementary Figure S1** Rarefaction curves and Venn analysis of soil samples. **Note:** The abscissa represents the amount of sequencing data randomly selected. The ordinate represents the observed Shannon index.

**
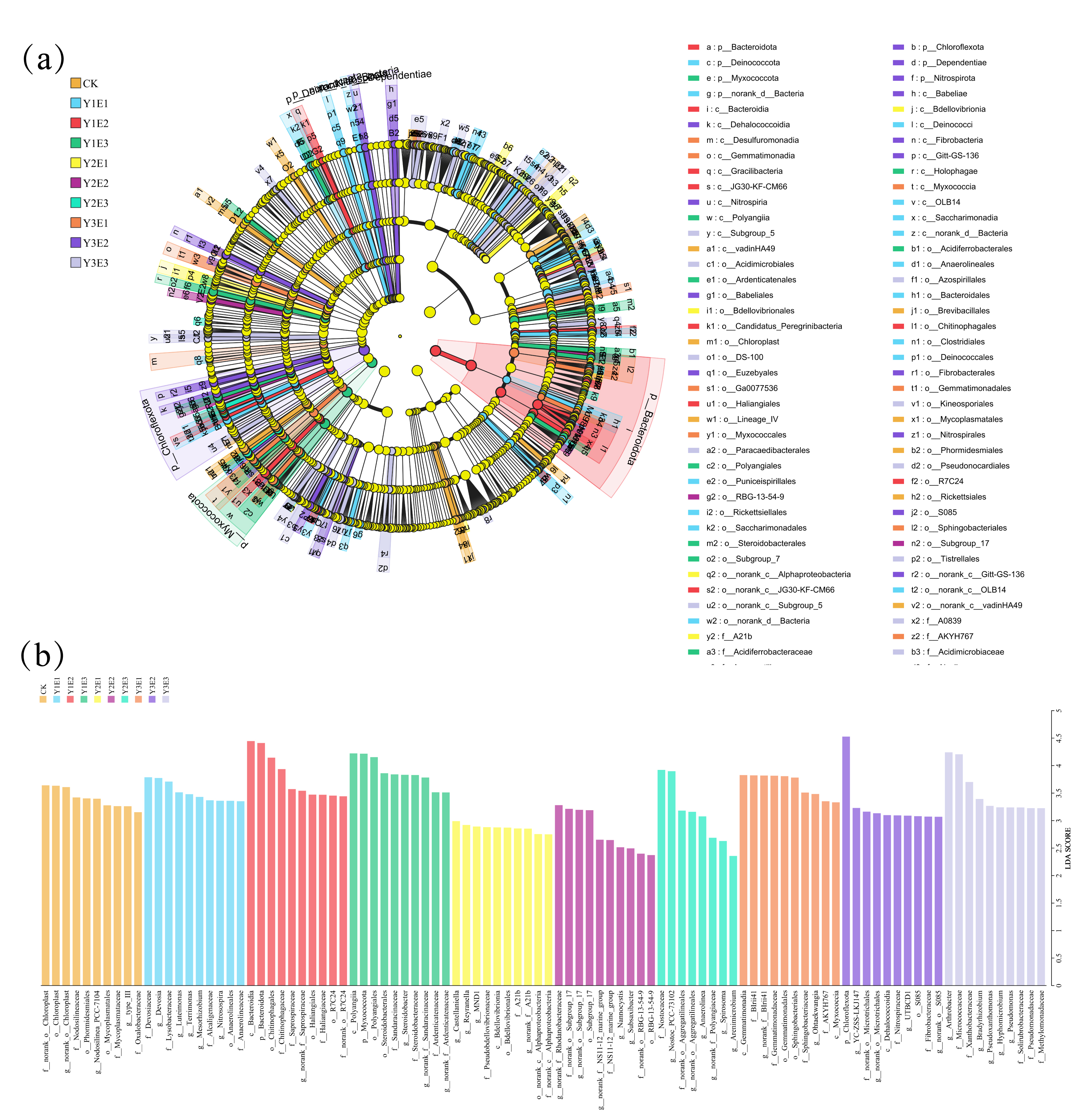
**

**Supplementary Figure S2** LEfSe analysis of soil bacterial communities across different treatments. **Note:** LDA > 2.5

**
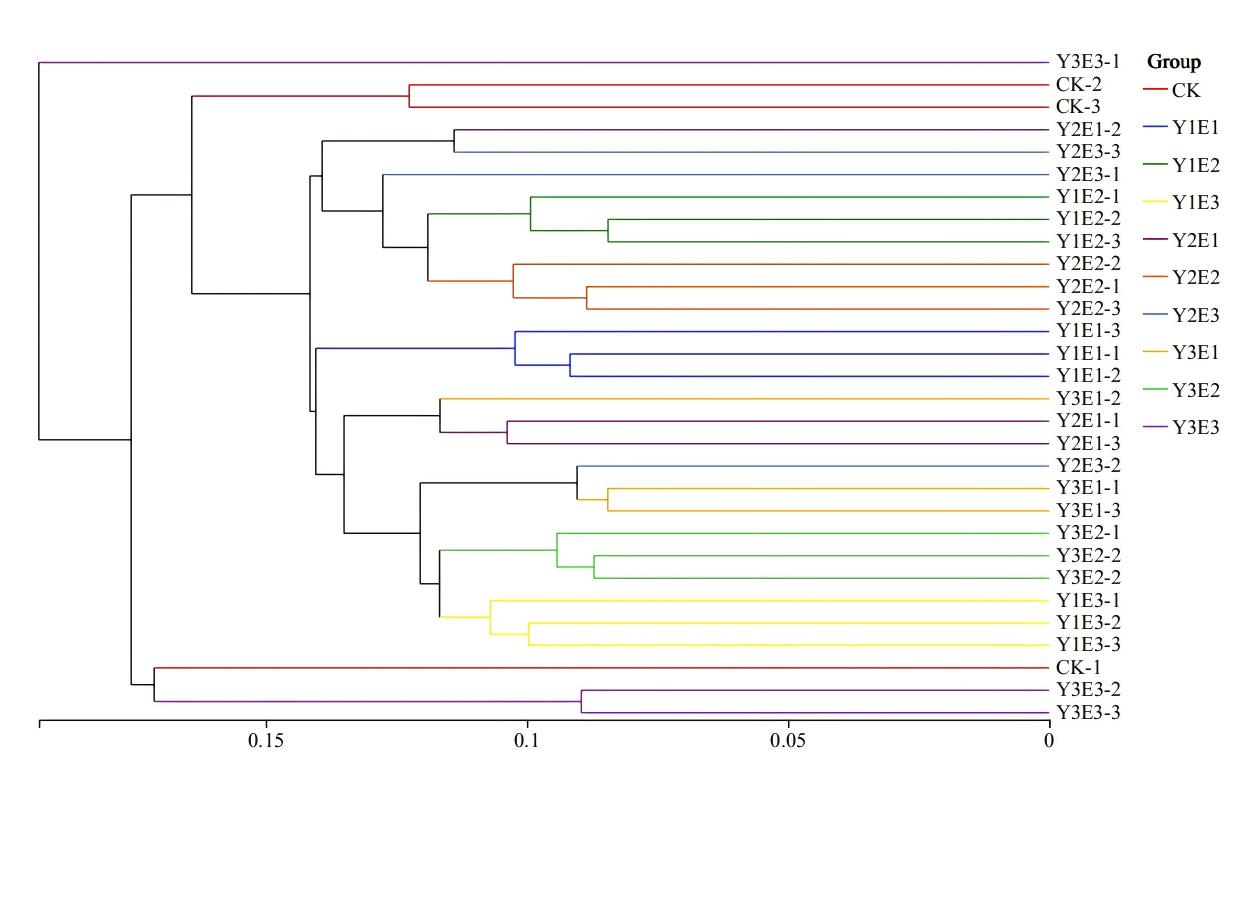
**

**Supplementary Figure S3** Clustering analysis of soil bacteria across different treatments.
